# Less is more: Allele-specific removal of dysfunctional RYR1 channel subunits

**DOI:** 10.1016/j.omtn.2024.102301

**Published:** 2024-08-30

**Authors:** Derek Sun, William R. Lagor

**Affiliations:** 1 Department of Integrative Physiology, Baylor College of Medicine, Houston, TX 77030, USA

## Main text

Ryanodine receptor isoform 1 (RyR1) is the essential calcium release channel in the sarcoplasmic reticulum (SR) of skeletal muscle linking membrane depolarization to muscle contraction. Mutations in RYR1 cause a range of diseases collectively referred to as RYR1 myopathies. Features of RYR1 myopathies include muscle weakness, fatigue, muscle pain, joint and skeletal abnormalities, breathing problems, cramps, and difficulty walking. In disorders with malignant hyperthermia susceptibility (MHS) , life-threatening hypermetabolic episodes can be triggered by exertion, heat stress, or anesthesia. In the current issue of *Molecular Therapy Nucleic Acids*, Beaufils et al.^1^ develop a new approach to correct dominantly inherited central core disease (CCD) with MHS, taking advantage of patient-derived myoblasts. Rather than focusing on the disease-causing mutation itself, the authors direct Cas9 and a pair of guide RNAs (gRNAs) to co-inherited single-nucleotide polymorphisms (SNPs) in non-coding regions. This enabled allele-specific removal of critical exons, which decreased mutant RYR1 protein subunits and restored channel function.

The very large size of the RYR1 coding sequence (15 kb) makes it a major challenge for gene therapy. Four RYR1 subunits assemble into a homotetramer to form the active channel. Even if an RYR1 transgene could be delivered, it is likely that new pathology would result from supraphysiological expression. As such, it is an ideal candidate for gene editing. Recently, Godbout et al. showed that prime editing could correct the T4709M mutation associated with a recessive form of the disease.^2^ Although precise sequence changes were installed, phenotypic correction of calcium handling was not assessed. While an important demonstration for recessive RYR1 myopathy, approaches like prime editing must be customized to each private mutation—a significant development and regulatory hurdle.

In contrast, dominantly inherited RYR1 myopathies present unique opportunities for treatment. Mice with heterozygous deletion of RYR1 show no discernible pathology, suggesting that half-normal levels of RYR1 protein are functional and well tolerated. Even a moderate reduction in the abundance of mutant RYR1 transcripts could have an outsized effect on function—by allowing for a larger proportion of channels with four normal RYR1 subunits. Previous studies by Loy et al. showed that allele-specific silencing of RYR1 mRNA is possible in the I4895T and Y524S mouse models of CCD and MH, respectively.^3^ Electroporation of allele-specific small interfering RNA (siRNA) into the flexor digitorum brevis muscles of the mouse footpad effectively rescued calcium handling defects.

In this work, Beaufils et al. are advancing a potential treatment for dominant RYR1 myopathy using CRISPR-Cas9 gene editing by targeting immortalized human myoblasts with the p.Y4796C mutation. This variant causes CCD and MHS due to increased calcium leak from the SR.^4^ Instead of targeting the mutation directly, the authors identified heterozygous SNPs from patient sequencing data that created a unique “NGG” protospacer adjacent motif for Cas9 cutting. The optimal pair of gRNAs successfully removed a 3,691 bp fragment of the pathogenic allele. The timing of editing was controlled using a “killer” lentivirus with a gRNA to self-inactivate Cas9. Functional analysis of edited myotube clones showed reduced mutant transcript levels (50%–33%) and normalized calcium responses and caffeine sensitivity. They also developed an open-source script, “CutOneStrand,” for efficient SNP screening and identified 571 dominant genes that could be similarly targeted.

A noteworthy advance of the paper is the concept of using co-inherited SNPs to disrupt the expression of a pathogenic allele. In the case of RYR1, this could enable the same pairs of gRNAs targeting common SNPs for gene therapy of numerous different mutations. Since the mechanism involves introduction of a frameshift and nonsense-mediated decay of the edited mRNA, the mutations do not need to reside in the 3.7 kb region but could be anywhere in RYR1. This could make editing reagents more generalizable to a broader group of patients. Clinical application would require careful sequencing and SNP phasing to identify the exact patients who would benefit.

Beaufils et al. show valuable proof of concept that allele-specific deletion of RYR1 could be a viable approach for dominant RYR1 myopathies. It is important to recognize that RYR1 is a four-subunit homotetramer. Therefore, the specific threshold of disease correction likely depends on the relative number of RYR1 channels that contain a mutant subunit. This may vary by the specific mutation in terms of mRNA and protein stability, as well as the mechanism of how channel activity is altered (i.e., increased or decreased calcium leak, heat sensitivity, etc.). Likewise, further work will be needed to confirm that 50% expression of normal RYR1 is truly benign. Although pathology is not readily evident in RYR1 heterozygous mice, this may not be the case in humans, where the time span covers decades rather than months of life.

There are also important caveats. The paper uses single-cell-derived myoblast lines to assess the molecular and phenotypic consequences of RYR1 editing. This provides clear information about calcium handling, the first such demonstration for RYR1 editing. However, it is also a best-case scenario, which would not progress to completion *in vivo*. A key question is what proportion of alleles in skeletal muscle would contain the desired deletion. Many edited alleles may have a small insertions or deletions (indels) at either cut site, which would preclude excision. In addition, there would likely be undesired on-target edits, including larger deletions, inversions, and chromosomal translocations. Off-target editing is also a concern with any genome editing approach. Although no edits were found at predicted sites, more comprehensive unbiased assays would be needed.

Creating a human gene editing therapy for RYR1 with allele-specific deletion would have to overcome additional hurdles. Broad and efficient delivery of Cas9 and gRNA to the entire musculature will be required. Although this is possible with adeno-associated virus (AAV), the field is currently grappling with the risks of very high doses—including liver injury, thrombotic microangiopathy, dorsal root ganglia toxicity, and myocarditis. Likewise, persistent expression of Cas9 in skeletal muscle is also a concern. Hakim et al.^5^ found that AAV delivery of CRISPR-Cas9 to dog models of Duchenne muscular dystrophy produced a Cas9-specific cytotoxic T cell response that eliminated dystrophin-positive fibers. Most humans also have pre-existing immunity to the commonly used Cas9 nucleases, making it important to use transient delivery. Beaufils et al.’s “killer gRNA” is an intriguing solution, but further work would be needed to optimize the timing and efficiency within a translatable delivery platform.

**Figure 1:**
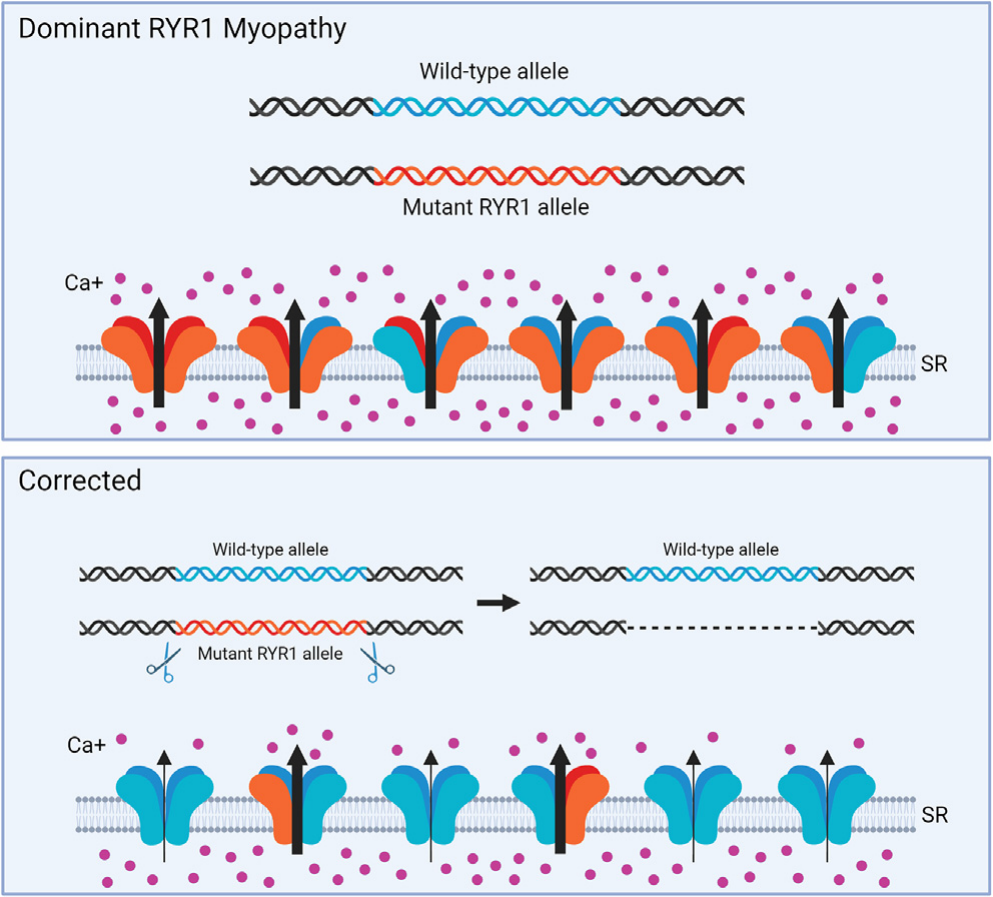
Gene editing strategy to correct an autosomal dominant RYR1 myopathy The p.Y4796C mutation in *RYR1* encodes dysfunctional channel subunits (orange) that result in excessive Ca^2+^ leak from the SR to the cytosol. Gene editing with CRISPR-Cas9 removes a large fragment of the p.Y4796C mutant allele by cutting at co-inherited SNPs. Following editing, myofibers express a larger proportion of normal RYR1 channels (blue) lacking the dysfunctional subunits, preventing excessive Ca^2+^ leak and hypersensitivity to caffeine.

In summary, Beaufils et al. offer valuable hope for the RYR1 community, with an exciting new approach for allele-specific removal of dominant RYR1 mutations (Figure 1). Their strategy involves non-homologous end joining, an efficient DNA repair pathway that is active in all skeletal muscles. In addition, the concept of targeting non-coding common SNPs is innovative and could be used to produce reagents that are generalizable to many patients. This is important for rare to ultra-rare diseases. Lastly, it is possible that upcoming advances with viral vectors or nanoparticles will finally crack the problem of transient delivery of CRISPR-Cas9 to the musculature. We look forward to seeing RYR1 editing moved into animal models and, ultimately, into humans. In the case of dominant RYR1 myopathies where a disease-causing channel subunit can be eliminated, sometimes “less is more”!

## Declaration of interests

The authors declare no competing interests.
